# Meeting Reports

**DOI:** 10.1155/S1463924679000778

**Published:** 1979


### Meeting Reports

### Summer School on Automatic Methods of Analysis

During the week of 9-13 July 1979, the first Summer School on Automatic Methods of Analysis was held at the University College of Swansea, Wales. The course was sponsored by the Chemical Society and organised by Dr. J. Betteridge of the college and Dr. P.B. Stockwell and Mr. D. Porter of the Laboratory of the Government Chemist.

The format of the course was based on a series of lectures in the mornings followed by informal tutorials and instrument demonstration sessions in the afternoons. Several international visitors gave lectures on topics ranging from compact flow injection analysis apparatus to the computer control requirements of very large instruments such as multi-laser systems.

To open the course, Dr. J. Foreman of the National Physical Laboratory gave a discourse on the manager's problems with the introduction of automation into industrial laboratories. The implications of different staff requirements, the purchasing and/or manufacturing of equipment and the impact on the customer of new analytical methods were discussed. Dr. F.L. Mitchell spoke on the introduction of automation to the clinical laboratory over the past 30 years. Illustrations were given of the earliest attempts to speed up analysis, and the latest multi-channel discrete analysers capable of providing results within seconds of the reaction commencing. A second lecture by Mitchell highlighted new techniques in clinical analysis, in particular the development of 'dry to the touch' analysis by Kodak. This has applications in rapid on-the-spot assays of biological fluids for sugars, urea etc.

J. Ruzicka, of the Technical University of Denmark, discussed the concepts of flow injection analysis (FIA) in which the sample is injected directly into a flowing reagent stream without air segmentation. The control and extent of dispersion in the flowing stream is of prime importance in fields such as measurement of pH, spectrophotometry and titrimetry. Some details of FIA applications using micro ion-selective electrodes for the determination of pH, pCa and nitrate were given by E.H. Hansen. Very short analysis times (of the order of 30 seconds) are possible with these systems. Ruzicka, Hansen and co-workers have been in the fore-front of development work in FIA, which has culminated in the marketing of commercial apparatus by Bifok of Sweden.

Modern electronics is playing an ever increasing role in chemical analysis, as evidenced by the inclusion of micro-processors into a wide range of instruments. H.L. Pardue of Purdue University, USA presented a survey of the users to which the 'new electronics' can be put in the field of improving analytical efficiency. He suggested that analytical chemists should consider learning basic electronic principles so that they may more easily converse with electronic engineers involved in construction of automated equipment. Dr. J. Betteridge elaborated further on the functions and advantages of micro-processors in modern technology and how they are capable of being used in applications such as replacement of relays and timers, controlling the operating sequence of equipment and performing computations. A paper by P.B. Stockwell outlined how the requirements of computer facilities at the Laboratory of the Government Chemist have altered over the past five years with the increasing usage of microprocessors in instruments. He predicted that, in the future, less direct data acquisition from apparatus would be performed by a central computer – its main functions would be to provide a central registry of programs and data, good data base management, better response to users, and availability of graphics and other peripheral units. When specifying and choosing a computer analysts must be assured that they can communicate and have control over the unit; not that it is a 'black box' to be avoided at all costs.

D.R. Deans of ICI Petrochemicals UK discussed the arguments for and against automation and outlined the criteria for introducing successful automation – these include the necessity to aid both the operator of the instrument and the user of the results, a good fail safe design and the ability to manually intervene in an operating system. A practical application of automation in the petrochemical industry was illustrated by reference to the gas chromography of complex mixtures. Flow switching is used to improve gas chromatographic separations by heart cutting and/or backflushing.

D.G. Porter discussed many of the aspects related to the design and construction of an automatic analyser in the users own laboratory. The requirements of the particular analysis and the necessary resources such as suitable staff, equipment, components and workshop facilities were considered in detail. It was stressed that it is vital for members of any automation groups to be fully aware of the concepts of automation and be able to convince prospective users of the benefits to be gained.

The functions of a recently introduced complex instrument for performing automatic single test analysis was described by H. Bartels of Ciba Geigy, Switzerland. The three linked stations of this Mettler system are capable of sample preparation including grinding of solids, extractions, dilution and titration to any specified end point. Many different types of analyses are capable of being carried out by suitable entry of the requirements into the systems computer control.

An interesting and most enthusiastic speaker, M.B. Denton of the University of Arizona, USA gave two lectures related to current and future trends in automation, particularly in relation to spectroscopy and electronics. Much emphasis was placed on proper system optimization by automatic techniques, a good example being the systematic determination of the best operating condition for atomic absorption – atomic emission spectrometers. It was predicted that the rapid advances being made in the micro-electronics industry will in future mean increased use of automated laboratory techniques with greater capabilities and flexibility than currently available.

Approximately 15 commercial instrument companies provided equipment and technical staff for demonstration purposes for the duration of the course. Although some operating difficulties were occasionally experienced, the opportunity for discussions with several manufacturers of automated equipment was welcomed. The success of this course should give an impetus for more companies to exhibit their products at future Automation Schools so as to give a much wider spectrum of the available equipment. The display of some automated equipment constructed by ICI Petrochemicals Ltd. and by the Laboratory of the Government Chemist could be extended further in future courses, for example, a photographic display of equipment constructed 'in house' by several organisations would prove valuable in demonstrating to participants that the development of automation is limited only by their ingenuity.

The tutorial sessions provided a valuable forum for the participants to discuss their particular automation problems and for the lecturers to explain in greater detail some of the points raised earlier. A course problem was set for each of the small tutorial groups, the objective being to develop arguments and define strategies to be adopted in the application of automation to the determination of tar, nicotine and carbon monoxide levels produced by cigarettes. The ideas developed during the discussions were an indication that total system automation rather than the use of sole automated techniques played a prominent part in the considerations. The underlying theme of several lecturers had been that the complete analysis from initial sample preparation through to final reporting should be considered as one system rather than looking at a project in a piecemeal fashion. G. Copeland of the Laboratory of the Government Chemist finally gave an expose of how the problem had been tackled and solved at the establishment.

It is my belief that the great majority of participants considered that this course was of considerable value in the dissemination and exchange of philosophies and ideas on automatic methods of analysis. Lessons learned in this initial venture should be put to good use in organising an even more successful school next year.

*B.S. Magor*

### Analysis '79

'Automation in Industrial and Clinical Chemistry' was the title of a symposium held on 16-18 July 1979 at the City University, London. It was organised by Scientific Symposia Ltd and sponsored by the Journal of Automatic Chemistry. Over the three days a wide range of analytical topics were covered on both industrial and clinical chemistry in the form of keynote addresses and lectures by speakers from America and Europe. A large number of delegates attended the conference, mainly from industry. The application of automation in industrial, clinical and academic environments was reviewed, with some emphasis on managerial and economic considerations and other factors that influence the organisation and implementation of automation.

The opening keynote address (1) by Professor Bonner Denton provided a comprehensive overview of the current trends of automation in chemistry. Professor Denton dwelt mainly on the factors that are of importance when considering automation: Is there a complete package available for the job? If not, what is the cost and effort required to modify a commercial package? Would it be better to make your own system using modules? He stressed the importance of considering not only the needs of today but also future requirements when developing automatic systems. Ideas for future automation and the further involvement of computers in the various stages of automatic analysis were also discussed. Professor Denton provided brief details of a computer language called CONVERS which he has designed for simplicity of use in compiling and executing programs [1].

Dr. L.B. Roberts' lecture (2) emphasised the importance of proper training for personnel who use automated systems. The professional analyst has a moral responsibility to provide accurate results, and to this end staff must know how to use their instruments. Operation and maintenance of analytical instruments are two separate jobs and it is necessary to have staff qualified for both. He considered it essential for manufacturers to provide training courses either on- or off-site. He also suggested that it might be useful to include a basic engineering/electronics course in a first degree. Maintenance contracts may be a prerequisite to the purchase of an instrument and often add considerably to its operating cost. The user should be provided with a maintenance manual for reference and basic in-house repairs, the manufacturer is often reluctant to supply this.

A report by Dr. D. Betteridge on the 1979 Chemical Society Summer School (3) which was held during the week prior to the symposium was a fitting subject to follow the lecture by Dr Roberts. The Summer School, held in Swansea and organised by Dr. Betteridge, Mr. D.G. Porter and Dr. P.B. Stockwell, was an experiment to provide education for automated analysis. The week provided a variety of theory, practical work and tutorial sessions. Several commercial companies had provided equipment and their expertise for the laboratory sessions.

Dr. Betteridge went on to chair the afternoon session which began with a second paper from Professor Denton(4). He gave a resume of the application of automation to spectroscopy. He commented on the use of computers and the importance of selecting a system that is economical, fast, reliable, simple and, most important, suitable to the user's needs.

Dr. J. Ruzicka (5) briefly described recent developments in flow injection analysis (FIA) with emphasis on the design of automated FIA systems and the use of stopped-flow, merging zones, extraction and scanning techniques.

An approach to kinetic analysis that allows observation times of up to 30 minutes at a throughput of 150 samples per hour has been developed at the Clinical Research Centre, London, in co-operation with Coulter Electronics. The system was described here by M. Snook (6). Prototypes of DACOS, the name given to the system, have been built and test data were present showing the photometric and overall performance of the system.

Day two of Analysis '79 commenced with Dr. Stockwell from the Laboratory of the Government Chemist discussing recent developments in automatic chromatography (7). He emphasised that where possible the system should be kept simple, although flexible enough to incorporate future requirements. The advantages and limitations of more recently introduced instruments were discussed.

A specialised application of conventional air-segmented continuous flow analysis was presented jointly by M. Stockley and R.J. Vincent (8), who described a method for the determination of sulphate in water using 2-aminopyrimidine. Results already obtained using this method compare favourably with those from other well-established techniques.

The second keynote lecture of the symposium, presented by Dr. F.L. Mitchell and entitled 'Concepts and trends of automation in clinical chemistry' (9), provided an insight into the development of automation in the clinical laboratory, the reasons for automation and the advantages and the problems that have arisen as a result. The problems of the small laboratory together with some solutions were surveyed. Dr. Mitchell drew attention to the fact that even in large laboratories the move now appears to be away from centralisation. Most laboratories do not have the required back-up for large automated systems, so it is natural to revert to using the smaller simpler instruments which perform one or two steps only but are reliable, eg, colorimeters. Layer chemistry was described and illustrated with the aid of Kodak's current work on the development of the Ektachem thin film technique.

The most obvious advantage to the clinician resulting from mechanisation is that larger numbers of analyses are made available more rapidly. The disadvantage is that clinicians who interpret the results assume an unlimited capacity for performing tests. Dr. Percy-Robb (10) outlined the consequences of automation such as the rising costs of equipment and of running the clinical laboratory. It must be remembered however, that to the doctor, automation has meant a marked improvement in the quality of results and in their comparibility between test stations.

Mr. Jones of Wessex Water Authority (11) talked about practical experiences in the development of a large purpose-built, 'automated' laboratory, and gave considerable importance to the possible long-term effects that automation may have on staff morale. He suggested ways of overcoming or preventing the problems.

T.M. Craig of Du Pont (12) presented a paper discussing a systematic approach for evaluating automation alternatives using cost benefit analysis. To illustrate the principles he used Du Pont's Value-in-use analysis, a computer-based cost-benefit analysis developed by himself.

Evaluation of an instrument is important because of the high cost involved when purchasing one. Dr. Roberts (13) drew on his own experiences at the Gartnavel General Hospital to illustrate the problems encountered during an evaluation. He stressed the responsibility and pressure on the evaluator in addition to the costs that are incurred, and he questioned whether the time taken from the start of the evaluation project – paper work starts many months before the machine actually arrives – to the final production of the report was completely justified.

Dr. Roberts was the chairman of the first session on the final day of the symposium. This was opened by a Keynote paper 'Developments in the philosophy and instrumentation for automated analyses' by Dr. Arndt of Mettler (14). Automation is accepted in all industries at the purely engineering level, but not at the social/political level despite the fact that the advantages of automation are borne out economically. Although industrial chemistry is done on a somewhat different basis to clinical chemistry, when the methods of analysis are broken down one finds that only a limited number of basic operations are involved which simplifies the job of automation. Progress has been made but only in small steps. It has not been hindered, as might have been expected, by economic recession because the stimulus – to reduce operating costs – is always present. One additional comment that Dr. Arndt made was that it must be remembered that ultimately the responsiblity will always lie with the analyst, not with the instrument or its manufacturer.

The enormous potential of radioimmunoassay and related analytical techniques in a clinical laboratory was expressed by Professor Landon of St. Bartholomew's Hospital. It ought to be possible to decide what parameter is to be measured and then to measure it. The usual approach, however, has been to find the parameters which are easy to measure and to automate their determination with little regard to the benefit. With RIA, it is possible to take the former approach.

Dr. Swann of the University of Nottingham (16) described his experience with the use of the Technicon InfraAlyzer for the determination of the protein content on cattle feed. This infra-red reflectance method is considerably faster than wet chemical analysis and therefore more acceptable to the farmer. The quality of results obtained so far are comparable to those of wet chemical analysis.

The next two papers dealt with problems in industrial analysis. Dr. Michel of Ciba-Geigy (17) described a hierarchical computer system, MIDAS, which collects raw data, computes results and provides control of the analytical instrument. A time-consuming procedure in the preparation of many samples for analysis is solvent extraction. Mechanisation of this process offers economy and safety to the chemist and is therefore worth consideration. Dr. Karlburg (18) discussed the development of a system based on the flow injection technique which has been constructed for pharmaceutical quality control purposes.

Dr. Coleman (19) from the National Physical Laboratory discussed an entirely different problem. With the advent of automatic methods of analysis there is a need to achieve accurate, unbiased and reproducible results. Instruments have to be tested during development and also require regular control and calibration procedures if they are to provide reliable and meaningful measurements. He discussed the production of certified reference materials for this purpose.

The final two papers of the symposium were complimentary. Both examined the subject of instrumentation for automation,Dr. Bierens de Haan (20) from the users' point of view and T. Craig (21) from a manufacturer's point of view. Clinical chemists believed in the early days of automation that all their problems would now be solved. Dr. de Haan said that this dream was soon shattered, and he felt that the fault lies both with the user and with the manufacturer. Users' demands are uncoordinated and often contradictory. There is not sufficient feedback from the user to the manufacturer. Better instruments will only be produced if these needs are well defined and fulfill the requirements of a large proportion of laboratories. T. Craig, speaking from the manufacturer's viewpoint, summarised the requirements for the successful development and commercialisation of an automated analytical instrument. Once the instrument is developed and on the market, it must be continuously up-dated to maintain the state-of-the-art technology. He added that the manufacturer also has the responsibility of training the user and providing a back-up service to the instrument once installed on a users' premises.

This symposium gave a useful review of the present situation of automation in laboratories and provided an opportunity for the interchange of ideas in the discussion periods and over lunch.

*J.S. Holme*

REFERENCE

[1] Tilden, Scott B. and Denton, M.Bonner, Journal of Automatic Chemistry, 1979, 1, 128.

BIBLIOGRAPHY

(1) Concepts in automatic laboratory systems: The trade-offs and rewards. Professor M Bonner Denton, The university of Arizona, Tucson, Arizona, USA.

(2) Training of clinical laboratory personnel in the use and maintenance of automatic systems. Dr. L.B. Roberts, Gartnavel General Hospital, Glasgow, UK.

(3) An experiment in education for automatic analysis: the 1979 Chemical Society Summer School. Dr. D. Betteridge, University College of Swansea, Swansea, UK.

(4) Case studies in laboratory automation. Professor M. Bonner Denton, The University of Arizona, Tucson, Arizona, USA.

(5) Recent developments in flow injection analysis. Dr. J. Ruzicka, Technical University of Denmark, Lyngby, Denmark.

(6) DACOS – a new approach to kinetic analysis. M. Snook, Clinical Research Centre, Harrow, Middlesex, UK.

(7) Recent developments in automatic chromatography. Dr. P. B. Stockwell, Laboratory of the Government Chemist, London, UK.

(8) Automated method for determination of sulphate in water. M. Stockley, Yorkshire Water Authority, UK and R.J. Vincent, Thames Water Authority, UK.

(9) Concepts and trends of automation in clinical chemistry. Dr. F.L. Mitchell, Clinical Research Centre, Harrow, Middlesex, UK.

(10) Medical consequences of automation in clinical chemstry. Dr. I.W. Percy-Robb, The Royal Infirmary, Edinburgh, UK.

(11) The cost benefits of automated analytical systems. J.G. Jones, Wessex Water Authority, Bath, UK.

(12) Economic techniques for evaluating automation alternatives. T.M. Craig, E I Du Pont de Nemours & Co, Wilmington, Delaware, USA.

(13) Evaluation of clinical laboratory equipment. Dr. L.B. Roberts, Gartnavel General Hospital, Glasgow, UK.

(14) Developments in the philosophy and instrumentation for industrial automated analysis. Dr. R.W. Arndt, Mettler Instrumente AG, Greifenesee, Switzerland.

(15) Automation of radioimmunoassay and related analytical techniques. Professor J. Landon, St. Bartholomew's Hospital, London, UK.

(16) Industrial application of automation with particular reference to the InfraAlyzer. Dr. H. Swann, University of Nottingham, School of Agriculture, Sutton Bonnington, Loughborough, UK.

(17) Automation in industrial chemistry through the use of a hierarchical computer system. Dr.G. Michel, Ciba-Geigy Ltd, Basle, Switzerland.

(18) Extraction in continuous flow systems with examples from pharmaceutical analysis. Dr. B. Karlburg, Astra Pharmaceuticals AB, Sodertalje, Sweden.
(19) Improved accuracy in automated chemistry through the use of reference materials. Dr. R.F. Coleman, National Physical Laboratory, Teddington, Middlesex, UK.
(20) Automatic systems – the users' needs. Dr. J. Bierens de Haan, Laboratoire Riotton SA, Geneva, Switzerland.
(21) Automatic Systems – the users' needs from a manufacturers' standpoint. T.M. Craig, EI Du Pont de Nemours & Co, Wilmington, Delaware, USA.

### Centrifugal analysers in clinical chemistry

*A symposium entitled 'Centrifugal analysers in clinical chemistry' was held in Southampton on 5-7 September 1979, organised by the Department of Chemical Pathology at Southampton General Hospital.*

The concept of centrifugal analysers and their application in clinical chemistry first appeared in the literature ten years ago with the first commercial instrument appearing in the United Kingdom in 1973. The technology has seen a great deal of change in the last few years, with the introduction of further commercial instruments to the market. The object of the Symposium was to review the concepts of centrifugal analysis as applied to the instrumentation available, to identify the way in which the analysers can be used in the laboratory and further, to try and identify what the future might hold.

In the first scientific session, Dr. Tom Tiffany reviewed the early work on centrifugal analysers and how this had been developed into the analysers currently available. Dr. Eisenwiener discussed the various approaches to photometric measurement and Dr. Burtis followed with a lecture on collection of photometric data and the use of computer software in centrifugal analysers. Dr. Oliver discussed the analysis of photometric error and how this can be used to design a means of monitoring performance. The session closed with a discussion by Professor Griffiths on how one should approach the evaluation of equipment. Professor Griffiths pointed out that decisions on the purchase of equipment are not based solely on technical specifications, but must include assessment of the ability of the instrument to fulfil the analytical role in the particular laboratory.

The next two sessions were devoted to the analytical techniques that may be employed on the centrifugal analyser. After an introduction to immunoassay by Dr. Edwards, discussion passed to the use of kinetic immunoturbidimetry for the measurement of specific proteins (Dr. Deverill). This was followed by a lecture on the use of homogeneous and heterogeneous immunoassay for small molecules with several examples of EMIT chemistries being given by Dr. H Greenwood. The immunoassay session concluded with a comparison of the assessment of thyroid function using enzyme and radioimmunoassay techniques.

Initially centrifugal analysers were regarded as sophisticated kinetic analysers and the merits of kinetic as against equilibrium assays were discussed by Dr. Renoe. The use of kinetic assays for enzymes was illustrated by Dr. Skillen and followed by the measurement of substrates by Dr. Zeigenhorn. The merits of kinetic techniques for the measurement of analytes by non-enzymatic reactions were discussed by Dr. Ertingshausen; this was followed by a short paper on the use of bichromatic analysis.

The spectrum of application of methods to the centrifugal analyser having been discussed, attention turned in the fourth session to the role of the instrument in the laboratory. Professor Marks proposed a more discriminating approach to test requesting, and this was followed by a paper on the use of centrifugal analysers to cover the major laboratory workload by Dr. Renoe. Dr. Westwood explored the potential of centrifugal analysers in the pediatric laboratory. Mr. Saunders from the Welsh Office, described some techniques for assessing the costs of laboratory work. Finally Dr. Mitchell gave a very interesting paper on the alternatives to parallel fast analysers, describing a new type of sequential fast analyser allowing complete workload flexibility.

The fifth and final scientific session explored the future of centrifugal analysers. Dr. Tiffany talked about the application of fluorescence measurement and Dr. Renoe described the use of a modified centrifugal analyser for laser nephelometry. Dr. Burtis described the potential for the technique of dynamic reagent injection. Dr. Greenwood described some interesting work of the use of homogeneous enzyme immunoassays for the measurement of specific protiens. The problems of temperature validation and a possible solution was discussed by Dr. Price. This session closed with two short papers on haematological applications.

The meeting was supported by demonstrations of the I.L. Multistat III Centrifichem 500, Rotochem IIa, Cobas Bio and ENI Gemeni centrifugal analysers.

The speakers have all provided a written text based on their contributions that the editors hope will provide a book describing the current "state of the art" regarding centrifugal analysis. The proceedings will be published by Holt Saunders in the early part of 1980.

*C.P. Price*
*K. Spencer*

### Clinical applications of HPLC, GC and MS

*Clinical Research Centre Symposium No. 1: Current Developments in the Clinical Applications of HPLC, GC and MS was held on May 30 – June 1 1979 and was organised at the Clinical Research Centre, Harrow, Middlesex, UK.*

This meeting provided opportunities for interested clinical chemists to learn more about the potentials of the applications of the three instrumental approaches to clinical analyses. While these methods are not yet widely used in the routine clinical laboratory, it was clearly pointed out by several speakers that they often enable difficult problems of general biomedical importance to be solved when solutions are not attainable with other methods.

The meeting opened on the afternoon of Wednesday 30 May with a session entirely devoted to HPLC. Four important papers were presented involving biological sample preparation (C.K. Lim, Harrow, UK), column switching techniques (J.F.K. Huber, Vienna, Austria), the basic aspects of soap chromatography (M. Gilbert, Edinburgh, UK) and ion-pair HPLC of drugs, metabolites and photochemicals (E. Tomlinson, Ohio, USA). The following day more applications of HPLC were discussed in the morning session: UV profiling of serum (Ph. Brown, Rhode Island, USA), investigations on bile pigments (C.H. Gray, Harrow, UK), peptides (W. Richmond, Harrow, UK) and, to conclude, a detailed and comparative description of all interfaces developed for online HPLC/MS (Games, Cardiff, UK).

A rather special meeting took place on Thursday afternoon when a number of short, 15 minute, oral communications were given exclusively by representatives of different commercial companies: Whatman Inc, Infotronics (UK) Ltd,

Spectra-Physics Ltd, Pye Unicam Ltd, Waters Associates Ltd, Shandon Southern Products Ltd, Technicon Instruments Co Ltd, V.G. Micromass, Finnigan Instruments Ltd, Packard Instruments Ltd, and Hewlett Packard Ltd. All presented industrial developments in analytical instrumentation with some relevance for clinical chemistry applications. These discussions served to reinforce the demonstration and exhibits also presented throughout the meeting by most of these companies.

The third day of the meeting started with an excellent review of the use of stable isotopes in biomedical research (P.D. Klein, Argonne, USA). In the second paper the philosophy of 'definitive' and 'reference' methodology, for which isotope dilution mass spectrometry is presently most indicated, was discussed (A.M. Lawson, Harrow, UK). The two following papers involved studies on respiratory gas analyses (D. Halliday and J.G. Jones, Harrow, UK). GC-MS applications in clinical pharmacology in general (G.H. Draffam, Edinburgh, UK) and the elucidation of the metabolism of the drug α-Methyldopa in the perinatal period (K.D.R. Setchell, Harrow, UK) terminated the session. In the afternoon in four contributions stress was again placed on the potential of GC and/or MS techniques. The first involved the characterisation of organic compounds of higher molecular weight using pyrolysis mass spectrometry (H.L.C. Meuzelaar, Utah, USA). This was followed by an extensive review of the diagnosis and investigation of organic acidurias by GC-MS (R.A. Chalmers, Harrow, UK). Another presentation demonstrated the practical aspects of capillary GC column operation and its utility for multicomponent analysis (C.H.L. Shackleton, Berkeley, USA). The last paper of the meeting concerned the complex problem of clinical steroid analysis (N.F. Taylor, Harrow, UK). Finally, Dr. F.L. Mitchell, Symposium Organising Chairman, closed the meeting with a few well-justified statements.

The Organising Committee has to be commended for their handling if this interesting conference. In addition, we had an unusual and fascinating experience on Thursday evening with a visit to the Magic Circle – a pleasant complement to the formal programme.

*A.P. de Leenheer*

### International conference on flow analysis

FA – Amsterdam, the International conference on Flow Analysis, was held on September 11 to 13 1979 at the vast and modern RAI conference centre on the southern outskirts of the city of Amsterdam. An informal get-together held on the evening of Monday, September 10, provided a pleasant opportunity for the participants to get to know one another and renew old acquaintances, by courtesy of Elsevier, the Dutch publishing group, at whose offices the reception was held.

The conference was opened on Tuesday morning by Professor G. den Boef of the University of Amsterdam, who also acted as chairman for the morning lecture session. The first paper was given by Dr. L.R. Snyder of Technicon Instrument Corporation, who reviewed existing methods of flow analysis and then went on to discuss the relative merits of air-segmented continuous flow analysis and flow injection analysis. He concluded by examining possible ways in which the best features of both types of system might be incorporated into a future design of hybrid analyser.

Professor J. Ruzicka of the Technical University of Denmark followed with a lecture on the theory of flow injection analysis and techniques for its application. He gave particular emphasis to the way in which reaction conditions and the extent of sample/carrier mixing could be manipulated by controlling the degree of dispersion in the flow system, and showed how a system with a high dispersion could be used to perform titrations by flow injection.

The first afternoon session was chaired by Dr. Snyder, and commenced with a lecture by Professor Ruzicka's co-worker, Dr. E.H. Hansen who described in detail some new analytical methods based on recently developed spectrophotometric and potentiometric flow-through detectors. This was followed by a paper by Dr. J. van den Berg of DSM Research who discussed various mathematical treatments available for the study of dispersion phenomena in reactors for flow injection analysis, including tubes and packed reactor cells.

The final lecture of the session was delivered by Professor N. Ishibasha from Kyushu University who overcame considerable language difficulties to describe a method for the determination of gallium by the fluorimetric detection of gallium – lumogallion complexes formed in a flow injection system.

After a short break the conference resumed for the final session of the day, which was chaired by Professor Ruzicka. The first talk was given by Ir. A. Scholten of Amsterdam University who described some photo-chemical reaction detectors for continuous flow (air segmented) systems. This was followed by a paper from Mr. B. Fields of University College, Swansea, describing a novel method for the multi-element analysis of trace metals, which relies on the fact that different metal ions undergo colour forming reactions with the same reagent at different pH values, and creates a linear pH gradient in the flow system to take advantage of this effect and thus separate the peaks derived from different metals.

Finally, Dr. K. Stewart of the U.S. Department of Agriculture's Nutrient Composition Laboratory described a microprocessor-controlled flow injection system capable of performing more than one type of analysis in the same flow system. Dr. Stewart stressed the need to maintain simplicity, especially in the microprocessor control system, and described a simple computer language which had been developed to enable technicians with no previous computer experience to use the system.

On Tuesday evening, the City of Amsterdam provided a reception for conference participants in the beautiful setting of the Amsterdams Historisch Museum. An official welcome was extended on behalf of the city, and everyone who attended had a most enjoyable evening.

The following morning, Dr. W.E. van der Linden took the chair for the first lecture session. Dr. K. Toth from the Technical University of Budapest gave the first lecture, a review of the various types of electrodes and associated equipment available for use in flow analysis and liquid chromatography, concentrating on detectors for continuous flow (air segmented) analysis, and including some new techniques developed in her laboratory. Dr. Toth's talk was followed by Dr. H. Reijnders of the Dutch National Institute for Public Health, who delivered a paper entitled "A modelling approach to establish experimental parameters of a flow-through titration."

He was followed by Dr. H. Poppe of Amsterdam University who described the characterisation and design of liquid phase flow-through detection systems, after which Dr. R. Tijssen lectured on the theory of dispersion and flow in liquid flow systems, and described a new theoretical model to describe the flow of liquids in helically coiled tubes.

When the conference resumed after lunch, Dr. Hansen took the chair for a lecture by Prof. G. Johansson of the University of Lund describing the applications of analytical enzyme reactors in flow systems. Prof. Johansson illustrated his talk with several examples, showing for instance how a reactor containing chemically bound urease could be used in a flow system to generate ammonium ions from samples of urea,enabling urea to be rapidly determined by measurement of the ammonium concentration with a suitable electrode.

The subject of enzyme reactors cropped up again during the first poster session, which followed Prof. Johansson's lecture. Of the nine displays, two, those of Dr. A. Ramsing of the Technical University of Denmark and Dr. M. Mascini of the University of Rome, described systems involving the use of enzyme reactors. Amongst the remaining displays, several interesting applications of flow systems were presented, including a review by Drs. Reijnders of available methods for the flow-through determination of sylphates, with a comparison of results obtained by the various methods, a microprocessor controlled system for automatic flow injection analysis by Mr. C.B. Ranger of Lachat Instruments Inc., and a method for the turbidimetric determination of sulphate by flow injection from Mr. Shirwan Baban of University College, Swansea.

A general discussion filled the remainder of Wednesday afternoon. This centred around the question of a suitable system of nomenclature to adequately describe various methods of flow analysis, and in particular to distinguish between methods involving air segmented flow, such as the Technicon AutoAnalyzer, and methods relying on a non-segmented flow. These two types have traditionally been known as continuous flow analysis and flow injection analysis respectively, but neither term adequately describes the system to which it relates since both involve the use of a continuous flow of carrier or reagent, and both involve the injection of a sample. However, despite a considerable amount of discussion no definite conclusions were reached.

The final social event of the conference took place on Wednesday evening with a boat trip around the canals of Amsterdam as far as Schiphol airport and back, during which a buffet meal was served on board. The trip lasted a total of three hours and provided the perfect end to the day.

On the final day of the conference, Dr. Toth chaired the first lecture session of the morning which opened with a paper from Dr. A. Jensen of the Danish School of Pharmacy that dealt with a flow injection system for the selective determination of magnesium, calcium and strontium which utilised the different rate constants for the formation of cryptate complexes of the different metals to enable selective determinations to be performed on a sample which contained a mixture of the various metal ions.

This was followed by a lecture by Professor H. Bergamin from Brazil who presented an interesting and extremely simple flow injection system for the simultaneous determination of nitrate and nitrite from water and soil samples.

The final lecture of the first morning session was given by Dr. Bo Karlberg of Astra Pharmaceuticals who described an ingenious method for performing solvent extraction in a flow injection system, as well as applications for the technique.

For the second lecture session of the morning, the chair was taken by Dr. B. Griepink of Utrecht University. The first paper by Dr. E. de Jong from Tilbury described the development of a new system of curve regeneration, providing a means of resolving more finely-spaced peaks than was previously possible and thus increasing throughput rates on an AutoAnalyzer system. After this, Dr. J.M. Reijn of Amsterdam University described some theoretical aspects of flow injection analysis, including injection effects and factors influencing the variance of peak heights. The final lecture of the morning was given by B. Pihlar from Ljubljana on flow analysis using cylindrical amperometric electrodes, and a method for the determination of complex cyanides.

In the afternoon the final poster session of the conference took place. Amongst several interesting presentations, Mr. O. Astrom of the University of Umea described a system for the determination of water in organic solvents, and Dr. Jack Betteridge and Miss Theresa Goad of University College, Swansea presented an interesting system for the

*(Continued on page 289)*

IF THIS IS WHAT YOU MEAN BY

AUTOMATIC CHEMISTRY

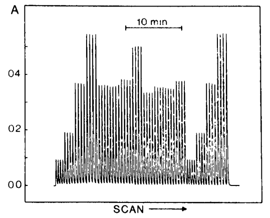

Routine analysis for phosphate in 20-times prediluted fertilizer samples with Flow Injection. From left to right is shown a set of aqueous standards (5, 10, 20, and 30 μg P—$PO_4$/ml) followed by 8 fertilizer samples and a second calibration run, all solutions were analysed four times. The sampling rate was ca. 120 determinations per hour.

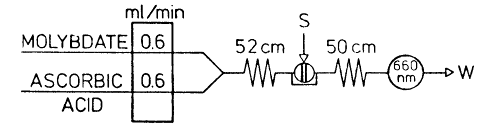

Flow Injection diagram for the spectrophotometric determination of phosphate. S — point of injection (30 μl); W — waste. All tube lengths are given in cm.

THEN YOU NEED

FLOW INJECTION ANALYSIS

The concept of flow injection

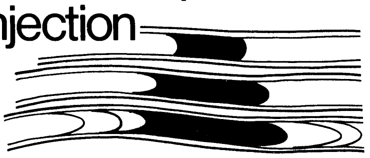

The flow injection method utilizes the principle of introducing a small sample volume into a stream of reagent in a controlled way. The degree of mixing and thus the degree of reaction is controlled by means of tube diameters and length and pumping rate.

Most types of titrimetric, turbidimetric, kinetic, photometric and potentiometric analyses are easily performed by FIA.

Various ways of treating samples, such as dialysis, dilution etc. can be performed.

**Applications include:—**

| | |
|---|---|
| * phosphate | 200-250 samples/hour |
| * nitrate | 90 samples/hour |
| * sulphate | 180 samples/hour |
| * ammonia | 60 samples/hour |
| * glucose | 120 samples/hour |
| * on-line dialysis | |
| * on-line gas diffusion | |
| * on-line titrations | |

For an obligation-free demonstration, advice on your analytical problem or literature on the tehcnique, please write or call today.

**EDT Research**
**14 Trading Estate Road**
**London NW10 7LU**
**Tel: 01-961 1477 TX: 8811955**

edt RESEARCH

